# Promoting clinical trials in pediatric IgA nephropathy through extrapolation: an artificial intelligence–enhanced literature review of the disease in pediatrics sponsored by the Kidney Health Initiative

**DOI:** 10.1007/s00467-026-07245-2

**Published:** 2026-03-08

**Authors:** David T. Selewski, Eleanor M. Kerr, Cecile Fajardo, Barbara S. Gillespie, Sai Prasad N. Iyer, Myda Khalid, Radko Komers, Koichi Nakanishi, Raoul D. Nelson, Jun Oh, Bonnie Schneider, Lokesh N. Shah, Nicholas J. Webb, Xuhui Zhong, Grace Squillaci, Mark D. Lim, Howard Trachtman

**Affiliations:** 1 Department of Pediatrics, University of Arkansas for Medical Sciences, Little Rock, AR USA; 2 Ripple Effect Communications, Inc, Rockville, MD USA; 3 Otsuka Pharmaceutical Development & Commercialization, Princeton, NJ USA; 4 International Study of Glomerular Diseases, Florence, MA USA; 5 SeaStar Medical, Denver, CO USA; 6 Riley Hospital for Children, Indiana University, Bloomington, IN USA; 7 Travere Therapeutics Inc, San Diego, CA USA; 8 Department of Child Health and Welfare (Pediatrics), Graduate School of Medicine, University of the Ryukyus, Ginowan, Okinawa Japan; 9 University of Utah Health, Salt Lake City, UT USA; 10 University Medical Center/at Hamburg, Hamburg/Eppendorf, Hamburg Germany; 11 IgA Nephropathy Foundation, Wall Township, USA; 12 Swedish Orphan Biovitrum AG, Basel, Switzerland; 13 Peking University First Hospital, Beijing, China; 14 Kidney Health Initiative, Washington, DC USA; 15 Department of Pediatrics, Division of Nephrology, University of Michigan, 1150 W Medical Center Drive, Med Sci 1/ARF 2511, Ann Arbor, MI 48109-1382 USA

**Keywords:** IgA nephropathy, Pediatrics, Clinical trials, Extrapolation, Literature review, Artificial intelligence

## Abstract

**Background:**

There is an urgent need to evaluate the efficacy of novel therapeutics that have been approved for use in adults with IgA nephropathy (IgAN) in pediatric patients with this primary glomerular disease.

**Methods:**

To explore the feasibility of deploying extrapolation to promote clinical trials in pediatric patients with IgA nephropathy (IgAN), an artificial intelligence (AI)–enhanced review of the published literature through 2023 was conducted to characterize the epidemiology and natural history of IgA nephropathy, summarize the clinical trial experience, and identify potential biomarkers for use in patient care and clinical trials.

**Results:**

Using Distiller SR® software to identify relevant articles and Elicit®, an artificial intelligence tool to extract content, 83 articles were included in the review. The clinical trial reports (*n* = 9) and biomarker studies (*n* = 33) were limited in scope. The natural history and epidemiology articles (*n* = 41) suggested that the severity of kidney histopathology at the time of diagnosis indicated a higher risk of disease progression while proteinuria < 0.5–1 mg/mg creatinine was associated with a more favorable prognosis.

**Conclusions:**

In conjunction with the similar biological basis of the disease in pediatric and adult patients, these findings may enable the use of extrapolation in the design of clinical trials in pediatric patients with IgAN.

**Graphical Abstract:**

A higher resolution version of the Graphical abstract is available as Supplementary information

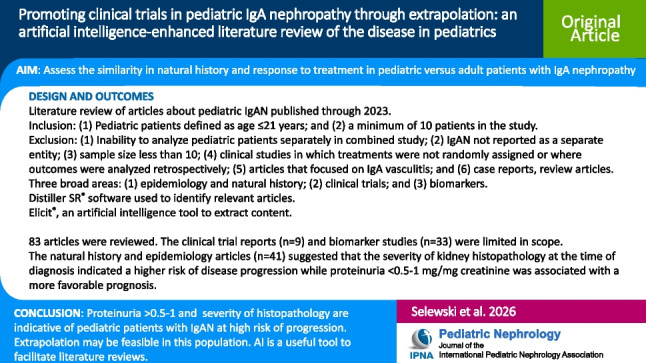

**Supplementary Information:**

The online version contains supplementary material available at 10.1007/s00467-026-07245-2.

## Introduction

IgA nephropathy (IgAN) is the most common glomerulonephritis worldwide. Although typified by a slow progressive course, a subset of adults and children with IgAN progress to chronic kidney disease (CKD) and kidney failure. The underappreciated and guarded long-term prognosis of patients with IgAN has been underscored by recent findings in the RADAR Registry in the UK. In that cohort, patients with biopsy-proven diagnosis of IgAN, proteinuria > 0.5 g/day, and eGFR < 60 ml/min/1.73 m^2^ (2299 adults and 140 children) progressed to kidney failure over 10–15 years of follow-up [1].

As a result of a Kidney Health Initiative (KHI) sponsored effort, a quantitative relationship has been established between the degree of reduction in proteinuria and a decrease in the likelihood of disease progression in adult patients with IgAN [2]. This led to acceptance of proteinuria as a reasonably likely surrogate endpoint for accelerated approval in randomized controlled trials (RCTs) in IgAN. Consequently, there has been a dramatic surge in the performance of clinical trials in adults with IgAN. This has led to the approval of a wide range of promising novel therapies including full approval for budesonide, sparsentan, and accelerated approval for iptacopan, atrasentan, and sibeprenlimab [3–7].

Considering the RADAR findings, it is essential to evaluate these treatments in pediatric patients with IgAN. In order to overcome the logistical barriers to the conduct of RCTs in pediatrics, regulatory authorities have advocated for the use of extrapolation, namely “an approach to providing evidence in support of effective and safe use of drugs in the pediatric population when it can be assumed that the course of the disease and the expected response to a medicinal product would be sufficiently similar in the pediatric [target] and reference (adult or other pediatric) population” (International Council on Harmonization E11(R1) guideline). Therefore, in order to assess the validity of applying extrapolation in pediatric IgA nephropathy, an artificial intelligence (AI)–enhanced literature review was conducted to (1) clarify the natural history of the disease in children and adolescents, specifically the disease course of current standard-of-care therapies, i.e., what happens to proteinuria and eGFR over time; and (2) identify features that may indicate pediatric patients with this glomerular disease who are at a high risk of disease progression.

## Methods

The Kidney Health Initiative (KHI) convened an international multidisciplinary work group including pediatric nephrologists, pharmaceutical company leaders, Food and Drug Administration representatives, and clinical trialists to address the gap in knowledge, namely, the natural history and response to treatment in pediatric versus adult patients with IgAN. The work group was charged to conduct a literature review of sufficient quality with a primary objective of determining the feasibility of applying extrapolation in the design of RCTs for pediatric IgAN.

Published literature about pediatric IgAN through 2023 was compiled using Distiller SR® software (Evidence Partners Inc., Ottawa, Ont., Canada). Articles were included if they satisfied the following qualifications: (1) inclusion of pediatric patients defined as age ≤ 21 years or the ability to analyze the findings in the pediatric patients separately if the report included both children and adults and (2) a minimum of 10 patients in the study. The team evaluated articles in three broad areas: (1) epidemiology and natural history; (2) clinical trials; and (3) biomarkers. Articles that combined adult and pediatric patients without the ability to analyze pediatric patients separately, that did not report IgAN as a separate entity, had a sample size less than 10, clinical studies in which treatments were not randomly assigned or where outcomes were analyzed retrospectively, case reports, review articles including Cochrane reviews, duplicate reports, and articles that focused on IgA vasculitis were excluded.

A detailed data extraction sheet was created for the AI application, and consensus on the relevant content for each of the three areas of interest was achieved. Relevant content, including clinical and laboratory data, was extracted using Elicit®, an artificial intelligence tool. The leadership team (DS and HT) reviewed the initial data extraction performed by the AI team to verify the accuracy of the data extraction, which was then validated and reviewed by the entire work group. A full list of the articles reviewed is provided in the Table.

This work was done under the aegis of the KHI and not a single academic site. In addition, the literature review was not considered to constitute human research because it involved analysis of publicly available material and no individual patient identifiers. Therefore, there was no formal review by an Institutional Review Board.

The literature search strategy and the full list of articles retrieved for review are available on request from the corresponding author.

## Results

After detailed review, 83 articles were included in the final analysis, including 9 on RCTs, 33 evaluating biomarkers, and 41 articles evaluating epidemiology and natural history. The published literature on RCTs in pediatric IgAN was limited in scope, enrolled mostly patients with mild-to-moderate disease, conducted primarily in Asian countries (*n* = 7), and evaluated treatments that are considered elements of the standard of care, corticosteroids (*n* = 6), or unconventional treatments such as tonsillectomy. The studies that addressed biomarkers enrolled relatively small numbers of patients and focused on histopathological features such as IgA or complement deposition and inflammatory cell infiltration, serum levels of galactose-deficient IgA1, IgA/C3 ratio, and oxidant stress. Most lacked long-term follow-up.

Regarding the natural history of pediatric IgAN, most studies originated from Asian countries (*n* = 27), included predominantly adolescents, and had modest numbers of patients at best and with limited follow-up. Few articles addressed the rate of spontaneous resolution or followed the participants into adulthood [8]. The use of renin-angiotensin aldosterone axis inhibitors consistently lowered proteinuria [9]. The number of patients who developed kidney failure was generally low and exceeded 10% in only three studies [9, 10]. Similarly, the occurrence rate of CKD was also quite low, ranging from 3 to 19% of the study sample.

Two observations were consistently documented in a subset of the articles. More severe structural abnormalities in the diagnostic kidney biopsy including presence of crescents, glomerular sclerosis, or interstitial fibrosis based on the pathologist’s description or MEST scoring were associated with a higher likelihood of persistent disease activity and/or progression [11–13]. In addition, in patients with proteinuria, urine protein:creatinine ratio less than 0.5–1.0 mg/mg had a more favorable prognosis [9, 14–16].

## Discussion

This KHI report is the first systematic review of the literature about pediatric IgAN that (1) utilized an artificial intelligence tool to facilitate data extraction from the relevant reports and (2) focused on key aspects of the disease with an eye to applying extrapolation as a tool to limit the scope of RCTs in children and adolescents with IgAN. The literature on clinical trials and biomarkers in pediatric IgAN is limited in scope and extent. In addition, in the published experience about long-term outcomes and response to treatment, only a minority of pediatric patients manifest progressive loss of kidney function because there is an underlying presumption that the prognosis is favorable. There is a paucity of data for pediatric patients with severe disease and a lack of information on long-term outcomes of change in proteinuria and eGFR that requires further prospective studies.

However, based on consistent data indicating elevated levels of antibodies to galactose-deficient IgA1 and circulating immune complexes in children with IgAN, the biological basis of the disease is likely to be comparable in pediatric and adult patients [17, 18]. The RADAR findings highlight the similar clinical course in patients with persistent disease across the lifespan. Thus, it may be feasible to apply extrapolation of findings in adult patients with IgAN to children and adolescents. This is consistent with the position that extrapolation from adults to pediatrics may be valid for patients with primary glomerular disease [19].

The literature review was more informative for identification of children at high risk of adverse kidney outcomes. The value of histopathological findings in predicting clinically significant disease progression in pediatric IgAN holds promise as a means of identifying patients at higher risk of disease progression and who should be prioritized for enrollment in clinical trials. This is feasible because pediatric patients with IgAN with significant clinical findings almost always undergo a kidney biopsy. Routine application of the Oxford (MEST) scoring system should enhance the value of the histopathological assessment and grading [20, 21].

Updating the review with key publications through 2025, the predictive value of histopathology in identifying high-risk patients is consistent with a recent meta-analysis of kidney biopsy findings in pediatric IgAN and has also been documented in children with IgA vasculitis [22, 23]. Prospective studies are needed to compare the value of histopathology findings with risk with the prediction formulas developed by Barbour et al. [24, 25]. It is reassuring that the prognostic value of a proteinuria cutoff of 0.5–1 g/g identified in this literature review aligns with findings in adult patients. It is congruent with a recent analysis of the trajectory of proteinuria observed over the first 2 years post-diagnosis in children and adults with IgAN enrolled in the CureGN cohort [22]. It is worth noting that adopting the lower limit, < 0.5 g/g, as the goal of treatment would be in alignment with the most recently updated Kidney Disease Improving Global Outcomes 2025 Guidelines for the treatment of patients with IgAN [26]. The updated pediatric version of the International IgA Nephropathy Prediction Tool requires further study. While the revised formula was useful in refining the prognosis in a large cohort of pediatric patients 1–2 years after a diagnostic kidney biopsy [25], it had suboptimal predictive value in a study of 472 Korean children, mean age 11.4 years, among whom 58 (14%) developed a 30% decline in eGFR or reached kidney failure over a median follow-up period of 48 months [27]. Finally, while there was evidence of differences in endoplasmic reticular, mitochondrial, and T-cell activity, the overall intra-renal transcriptomic signature is similar in adult and pediatric patients with IgAN, supporting the application of extrapolation [28].

The use of the AI tool to review the literature and extract relevant data was an important feature that facilitated the performance of this KHI-sponsored project. We recognize that AI is not a validated approach for this purpose nor is it a definitive analytical tool. In addition, its use in this context did not uncover new biological insights or predictive patterns. Future applications may include the potential to integrate multi-omics data or simulating trial outcomes.

There are several limitations to this study. The time period of the literature review, namely through 2023, antedates reports describing novel B-cell targeted therapies; however, these agents have not been evaluated in pediatric patients. Although the comprehensiveness of the literature review in pediatric IgAN was not confirmed by an independent search, the Distiller tool has been widely used in a broad range of medical topics relevant to pediatrics including serum iron and hemoglobin and Campylobacter infection [29, 30]. We did limited validation of the AI extraction tool and acknowledge that there may be some errors in the article summaries. Finally, the conclusions and the relevance to extrapolation are hypothesis-generating due to the reliance on a limited number of relatively small, heterogeneous clinical studies.

## Conclusion

Incorporation of an AI tool facilitated the performance of a literature review of IgAN in pediatric patients. Based on the similar pathophysiology of the disease and response to treatment of the disease, it may be reasonable to extrapolate adult data to pediatric patients with IgAN. The level of proteinuria after a trial of standard-of-care treatment and the severity of kidney histopathology findings may be useful measures to identify high-risk pediatric patients with IgAN who should be prioritized for enrollment in clinical trials evaluating the efficacy of novel therapeutic agents that target the immunological basis of the disease.

## Supplementary Information

Below is the link to the electronic supplementary material.

Graphical abstract (PPTX 76.5 KB)

## Data Availability

No new data were generated in this study. The list of articles reviewed is provided in the Table. A list of all articles retrieved for potential review is available on request to the corresponding author.

## Appendix. List of references included in the artificial intelligence-assisted literature review of pediatric IgA nephropathy

**Table:**

First author	Year	Journal	PMID number
Clinical trials (*n* = 9)			
Yoshikawa N	1999	JASN	9890315
Cano	2003	Clinical Nephrology	12940609
Chan J	2003	Pediatric Nephrology	2920628
Kawasaki Y	2006	Pediatric Nephrology	16932894
Hogg RJ	2006	CJASN	17699247
Yoshikawa N	2006	CJASN	17699253
Kamei K	2011	CJASN	21493743
Shima Y	2018	Pediatric Nephrology	29987456
Shima Y	2019	Pediatric Nephrology	30284023
Natural history/epidemiology (*n* = 41)			
Aoto	2022	Clin Exp Nephrol	35138499
Cambier	2020	Pediatr Nephrol	32444925
Coppo	2010	Kidney International	20200498
Coppo	2016	Pediatr Nephrol	27557557
Halling	2012	Nephrology Dialysis Transplantation	21750154
Enya	2020	Pediatrics International	32315477
Fabiano	2016	Jornal de Pediatria	28130969
Fu, Q-Y	2020	Nephron	26890857
Giani	1994	Renal Failure	7855318
Haas	2008	Nephrology Dialysis Transplantation	18263928
Higa	2015	Pediatr Nephrol	26238276
Hogg	1994	Pediatr Nephrol	8142218
Kamel	2016	PLoS ONE	26978656
Kamel	2011	CJASN	21493743
Kamel	2015	Pediatr Nephrol	25487669
Kang	2015	Pediatr Nephrol	25773534
Kawasaki	2004	Am J Nephrol	15550753
Le	2012	BMC Nephrol	23181565
Matsushita	2015	Clin Exp Nephrol	25800961
Mizerska-Wasiak	2017	Adv Exp Med Biol	27718216
Mizerska-Wasiak	2015	Adv Exp Med Biol	26269025
Mizerska-Wasiak	2021	J Clin Med	34640422
Murakami	1993	Nihon Jinzo Gakkai Shi	8107307
Nakanishi	2009	Pediatr Nephrol	18825420
Nozawa	2005	Clin Neph	16175941
Ronkainen	2006	Pediatr Nephrol	16838184
Ruggajo	2016	PLoS ONE	27092556
Selewski	2018	Kidney International Reports	30450464
Shima	2021	Pediatr Nephrol	33011820
Shima	2017	Pediatr Nephrol	27714465
Shima	2020	Pediatr Nephrol	31993782
Wu	2020	J Nephrol	32507961
Wu	2021	Clin Exp Nephrol	33620604
Wu	2020	BMC Nephrol	32611399
Wyatt	1995	Journal of Pediatrics	8523188
Yagi	2003	Clinical and Experimental Nephrology	14712355
Yoshikawa	1987	J Pediatr	3550023
Yoshikawa	1987	Clin Nephrol	3427831
Zhao	2021	Medicine	34032732
Zhuang	2022	Pediatr Nephrol	35347403
Biomarkers (*n* = 33)			
Beom Jin Lin	2009	Pediatric Nephrology	19066978
Seeman	2008	Kidney & Blood Pressure Research	18946224
Andreoli	1986	American Journal of Nephrology	3515942
Mizerska-Wasiak	2021	Cent Eur J Immunol	34764788
Berg	1993	Pediatric Nephrology	8476701
Kamano	2021	Clinical and Experimental Nephrology	33595730
Kawasaki	2017	Pediatrics International	27341677
Inaba	1996	Nephron	8730414
Hattori	1985	American Journal of Nephrology	4014324
Fabiano	2017	Pediatric Nephrology	28233100
Kawasaki	2014	Nephrology	24646214
Han	2012	Experimental and Therapeutic Medicine	22969955
Li	2019	Clinical and Experimental Nephrology	31468231
Hastings	2012	International Journal of Nephrology	22754697
Kawasaki	2018	Pediatrics International	29178575
Lang	2021	Translational Pediatrics	33880336
Baek	2018	Fetal and Pediatric Pathology	30273085
Pei	2016	Journal of the Renin-Angiotensin-Aldosterone System	27198042
Mizerska-Wasiak	2014	Pediatric nephrology (Berlin, West)	25549975
Suh	2011	Journal of Interferon and Cytokine Research	21214373
Shima	2011	Nephrology, Dialysis and Transplantation	20601366
Sato	2019	Pediatrics International	31237969
Topaloglu	2013	Clinical Kidney Journal	24175085
Mizerska-Wasiak	2022	Journal of Clinical Medicine	35268356
Toth	1998	Nephron	9807037
Sun	2015	PLoS ONE	25647400
Wasilewska	2011	Scandinavian Journal of Urology and Nephrology	21034351
Tang	2021	Clinical and Experimental Nephrology	32935202
Zhou	2023	Pediatric Nephrology	36102962
Urushihara	2015	Pediatric Nephrology	25523477
Watanabe	2001	Pediatric Nephrology	11465805
Wu	2022	Pediatric Nephrology	35697863
Cambier	2021	Kidney International	34756952
