# Supplementary material for: Promoting clinical trials in pediatric IgA nephropathy through extrapolation: an artificial intelligence–enhanced literature review of the disease in pediatrics sponsored by the Kidney Health Initiative
Source: Pediatr Nephrol. 2026 Mar 8;41(8):2481–7. doi: 10.1007/s00467-026-07245-2 (PMC13337918; doi:10.1007/s00467-026-07245-2)
Supplement: Supplementary file 1 — Graphical abstract (PPTX 76.5 KB) [file 467_2026_7245_MOESM1_ESM.pptx]

## Slide 1
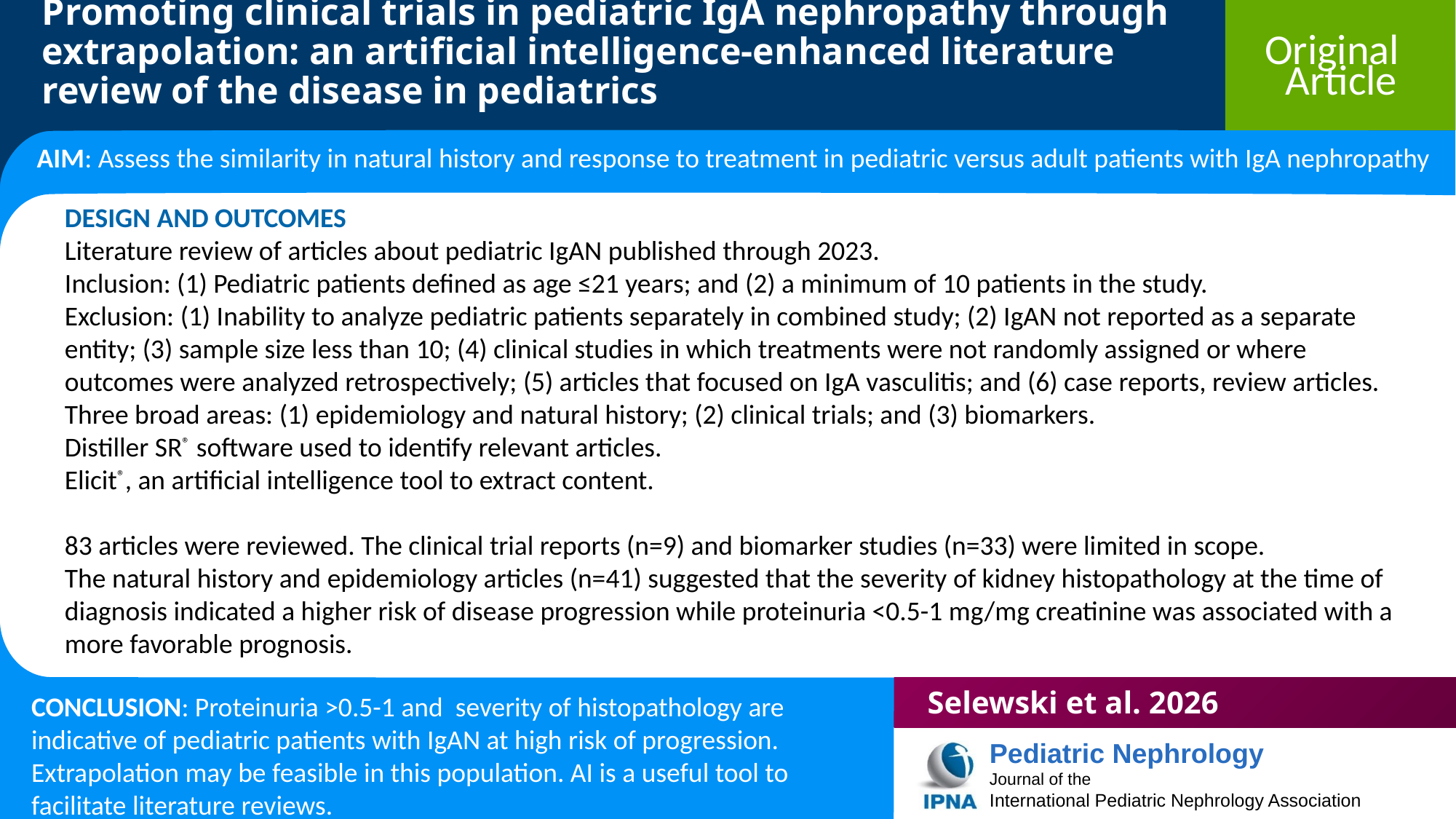

Promoting clinical trials in pediatric IgA nephropathy through extrapolation: an artificial intelligence-enhanced literature review of the disease in pediatrics
AIM: Assess the similarity in natural history and response to treatment in pediatric versus adult patients with IgA nephropathy
DESIGN AND OUTCOMES
Literature review of articles about pediatric IgAN published through 2023.
Inclusion: (1) Pediatric patients defined as age ≤21 years; and (2) a minimum of 10 patients in the study.
Exclusion: (1) Inability to analyze pediatric patients separately in combined study; (2) IgAN not reported as a separate entity; (3) sample size less than 10; (4) clinical studies in which treatments were not randomly assigned or where outcomes were analyzed retrospectively; (5) articles that focused on IgA vasculitis; and (6) case reports, review articles.
Three broad areas: (1) epidemiology and natural history; (2) clinical trials; and (3) biomarkers.
Distiller SR® software used to identify relevant articles.
Elicit®, an artificial intelligence tool to extract content.
83 articles were reviewed. The clinical trial reports (n=9) and biomarker studies (n=33) were limited in scope.
The natural history and epidemiology articles (n=41) suggested that the severity of kidney histopathology at the time of diagnosis indicated a higher risk of disease progression while proteinuria <0.5-1 mg/mg creatinine was associated with a more favorable prognosis.
Selewski et al. 2026
CONCLUSION: Proteinuria >0.5-1 and severity of histopathology are indicative of pediatric patients with IgAN at high risk of progression. Extrapolation may be feasible in this population. AI is a useful tool to facilitate literature reviews.
